# *Ex Vivo* Micro-Computed Tomography-based Three-Dimensional Modeling and Biomechanical Assessment of the Human Aortic Root

**DOI:** 10.21470/1678-9741-2025-0250

**Published:** 2026-09-08

**Authors:** Marien Lenoir, Tom Fringand, Isabelle Cheylan, Laurent Sabatier, Julien Favier, Loïc Macé

**Affiliations:** 1 Department of Cardiac Surgery, La Timone Hospital, Assistance Publique - Hôpitaux de Marseille, Aix Marseille University, Marseille, France; 2 Labotatoire M2P2, Centre National de la Recherche Scientifique, Centrale Marseille, Aix Marseille University, Marseille, France; 3 Centre National de la Recherche Scientifique, Centrale Marseille, Aix Marseille University, Marseille, France

**Keywords:** Aortic Root, Biomechanical Properties, Micro-CT, Aortic Valve.

## Abstract

**Introduction:**

Understanding the biomechanical behavior of the aortic root is essential for improving surgical planning and patient-specific simulations. Traditional tests such as planar biaxial testing are limited by small sample size and loss of three-dimensional (3D) structure. We propose a novel method combining static pressurization and micro-computed tomography (CT) imaging of intact human aortic roots to simultaneously obtain accurate 3D geometry and biomechanical data.

**Objective:**

To evaluate the mechanical properties of healthy human aortic roots under physiological pressure conditions.

**Methods:**

Ten aortic roots were collected from unused human donor hearts (non-cardiac death) with family consent. The roots were gradually pressurized from 20 to 120 mmHg in 20 mmHg increments and scanned at each step using high-resolution micro-CT. Measurements included diameter, stress-strain curves, and Young’s modulus.

**Results:**

3D reconstructions of all ten aortic roots were successfully performed. The mean donor age was 61.2 years (49.5 - 65.6). At 80 mmHg, the median diameter of the sinotubular junction was 32.2 mm (29 - 36). Stress-strain analysis revealed non-linear behavior, with significantly lower stiffness at low pressure (20 - 80 mmHg, median Young’s modulus 0.6 [0.5 - 0.8] MPa) compared to high pressure (80 - 120 mmHg, 3.2 [2.5 - 5.6] MPa, P = 0.001).

**Conclusion:**

This study demonstrated that ex vivo micro-CT combined with static pressurization can reliably evaluate the mechanical properties of healthy human aortic roots under physiological pressure ranges. The method provided high-fidelity 3D reconstructions and reproducible biomechanical metrics, consistent with existing literature. These results supply robust input for computational modeling and may help optimize rootsurgical procedure.

## INTRODUCTION

**Table:** 

Abbreviations, Acronyms & Symbols	
3D	= Three dimensional
BMI	= Body mass index
CT	= Computed tomography
FSI	= Fluid-structure interaction
IQR	= Interquartile range
STJ	= Sinotubular junction

To effectively address aortic root surgery, a thorough understanding of the structure’s physiology and biomechanics is essential^[1]^, a principle emphasized by Schäfers in his “10 commandments for aortic valve repair”. This requirement applies broadly to all aortic root diseases, including acute conditions such as aortic dissection.

Aortic root dilation is a serious condition, frequently associated with life-threatening complications such as dissection or rupture^[2]^. Surgical decisions have traditionally relied on maximal aneurysm diameter^[2]^. However, this approach may be insufficient, as recent studies highlight the potential value of additional parameters - including root geometry and rupture indices derived from finite element modeling - to refine risk stratification and guide surgical indication^[3-5]^.

Numerical simulations that integrate blood flow dynamics and tissue deformation - known as fluid-structure interaction (FSI) models - offer a promising way to improve our understanding of aortic root behavior^[6]^. These models reproduce cusp coaptation and assess the mechanical performance of the root under physiological conditions^[7,8]^.

Building such models requires precise input data, including:

• a high-resolution three-dimensional (3D) mesh of the aortic root,
• geometric dimensions (diameter, wall thickness, etc.),
• and mechanical properties (elasticity, stiffness).

Conventional mechanical testing methods, such as planar biaxial tests, are limited by their reliance on small flat samples and the assumption of tissue homogeneity. As such, they fail to capture the complex geometry and mechanical heterogeneity of the intact aortic root.

To overcome these limitations, we developed an innovative protocol combining static pressurization of intact human aortic roots with high-resolution micro-CT imaging. This approach enables simultaneous acquisition of anatomically accurate 3D geometry and detailed biomechanical data (stress-strain curves, Young’s modulus), while preserving tissue integrity.

The objective of this study is to characterize the biomechanical properties of healthy human aortic roots using this integrated methodology, and to provide physiologically relevant input data for future computational modeling.

## METHODS

### Ethical Approval

The study was approved by the Committee on Human Research at the French Agency of Biomedicine (PFFS 20-026). Aortic roots were obtained from unused donor hearts with non-cardiac causes of death, with family consent for research. The pulmonary valves were removed and cryopreserved as part of the standard tissue donation and processing protocol established by the French Biomedicine Agency. These valves are added to our institutional homograft bank and are later used for clinical procedures such as the Ross operation.

### Inclusion and Exclusion Criteria

Inclusion criteria were unused human donor hearts obtained with family consent, with non-cardiac causes of death and no macroscopic evidence of aortic root or valve pathology. Exclusion criteria were a known history of connective tissue disorders (such as Marfan syndrome, Loeys-Dietz syndrome, or Ehlers-Danlos syndrome), macroscopic calcification, or structural damage to the aortic root during retrieval.

### Preparation of Aortic Roots

Donor hearts were meticulously prepared in the operating room. Pulmonary valves were removed and cryopreserved, after which the aortic roots were harvested. This methodology was developed by one of us (L. Macé) on porcine aortic root. Particular care was taken to seal the coronary ostia using Prolene® 4/0 sutures to ensure no air leakage during pressurization. In our protocol, a single Frater stitch^[9]^ was placed, passing through the three nodules of Arantius. This technique creates central coaptation of the cusps and ensures valve competence during pressurization. A connector suitable for pressurization was securely attached to the aortic root, ensuring compatibility with subsequent procedures. The pressurization connector was systematically placed immediately after organ retrieval, following a standardized protocol. We preserved as much length as possible of the ascending aorta up to the origin of the brachiocephalic trunk. A Gardena®-type connector was then inserted into the aortic lumen.

To secure the seal, a 2-0 silk tie was wrapped and tightened around the aorta at the connector interface, ensuring a watertight closure of the root. This setup allowed stable pressurization without leakage during subsequent imaging.

Aortic samples were stored at +4°C in buffered saline solution between retrieval and imaging. No sample was stored for more than seven days.

### Micro-Computed Tomography Imaging

The prepared aortic roots were transported to the imaging facility in an isothermal tank to maintain optimal tissue conditions. Scanning was performed using a micro-computed tomography (CT) system calibrated to operate at a constant temperature of 24°C. The aortic roots were gradually pressurized using a syringe connected to a manometer. Imaging was conducted at incremental pressures of 20 mmHg up to 120 mmHg. Each pressure level required approximately three to four minutes of imaging time, during which air tightness was rigorously maintained to prevent deflation and ensure accurate results (Figure 1). The use of air as the pressurization medium provided high-contrast images, critical for detailed geometrical analysis.

**Fig. 1:**
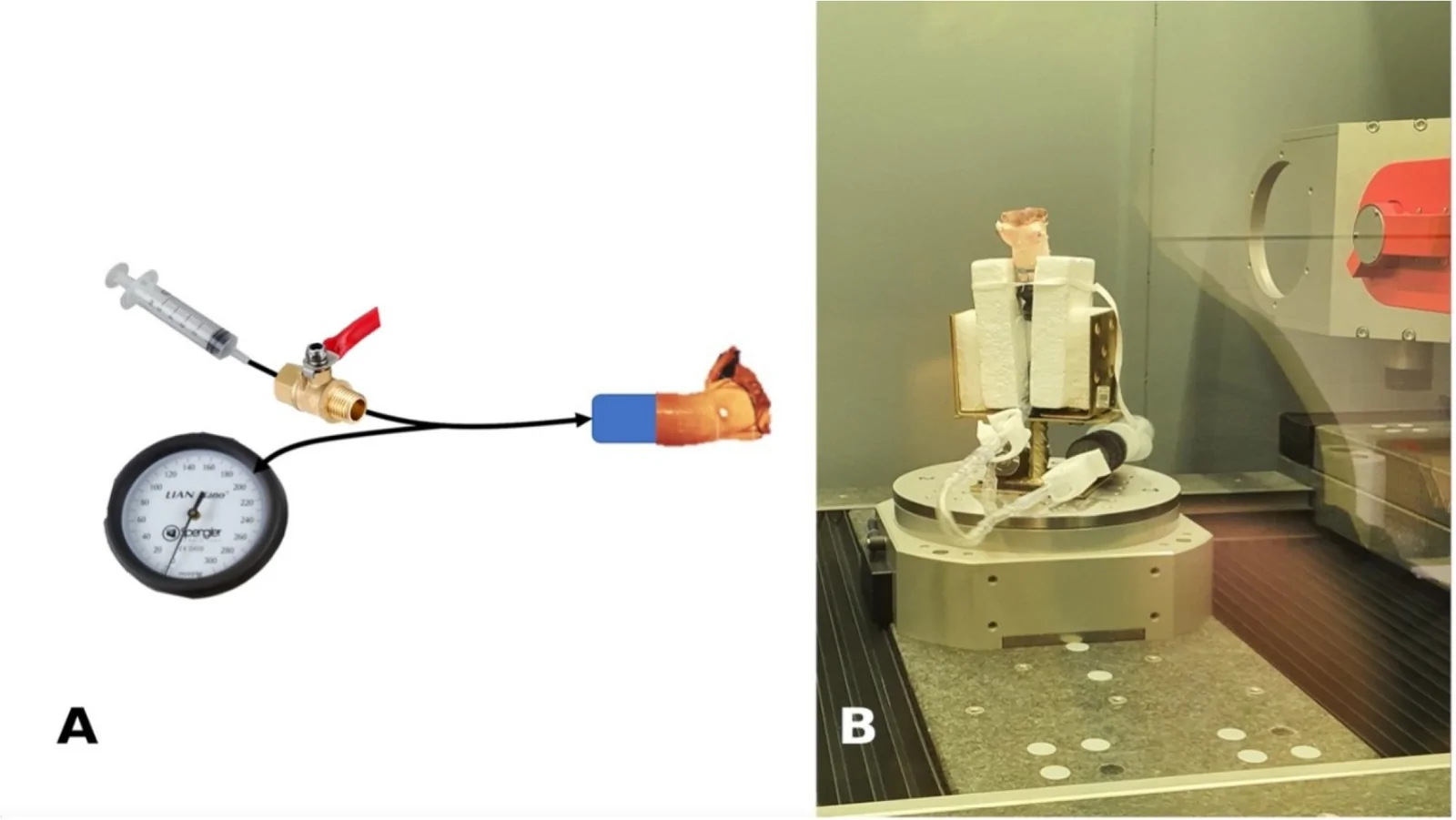
(A) Schematic picture of the main components of insufflation test equipment. (B) Image acquisition of the aortic root during micro-computed tomography.

### Micro-Computed Tomography Parameters

• X-ray source: sealed micro-focus tube, 150 kV, aluminum filter (1.2 mm)
• Acquisition settings:
  - Voltage: 100 kV
  - Current: 300 µA
  - Focal spot size: 20 µm
  - Voxel size: 50 µm
• Detector: amorphous silicon flat panel sensor
  - Pixel matrix: 1920 × 1536
  - Active area: 1874 × 1496 pixels
  - Pixel size: 127 µm
• Acquisition protocol:
  - One full rotation
  - 448 projections per rotation
  - Frame averaging over three images
  - Frame rate: 12.5 Hz
  - Image orientation: landscape
• Reconstruction method: Filtered back-projection

### Image Processing and Analysis

Micro-CT data were processed using Horos® software. Geometric landmarks, particularly at the sinotubular junction (STJ), were identified to ensure consistent measurements across samples. The cross-sectional area was calculated using a polygon tool, and the diameter was derived from the area using the formula π r^2^. This method provided accurate measurements of the STJ and other key regions of the aortic root (Figures 2 and 3).

**Fig. 2:**
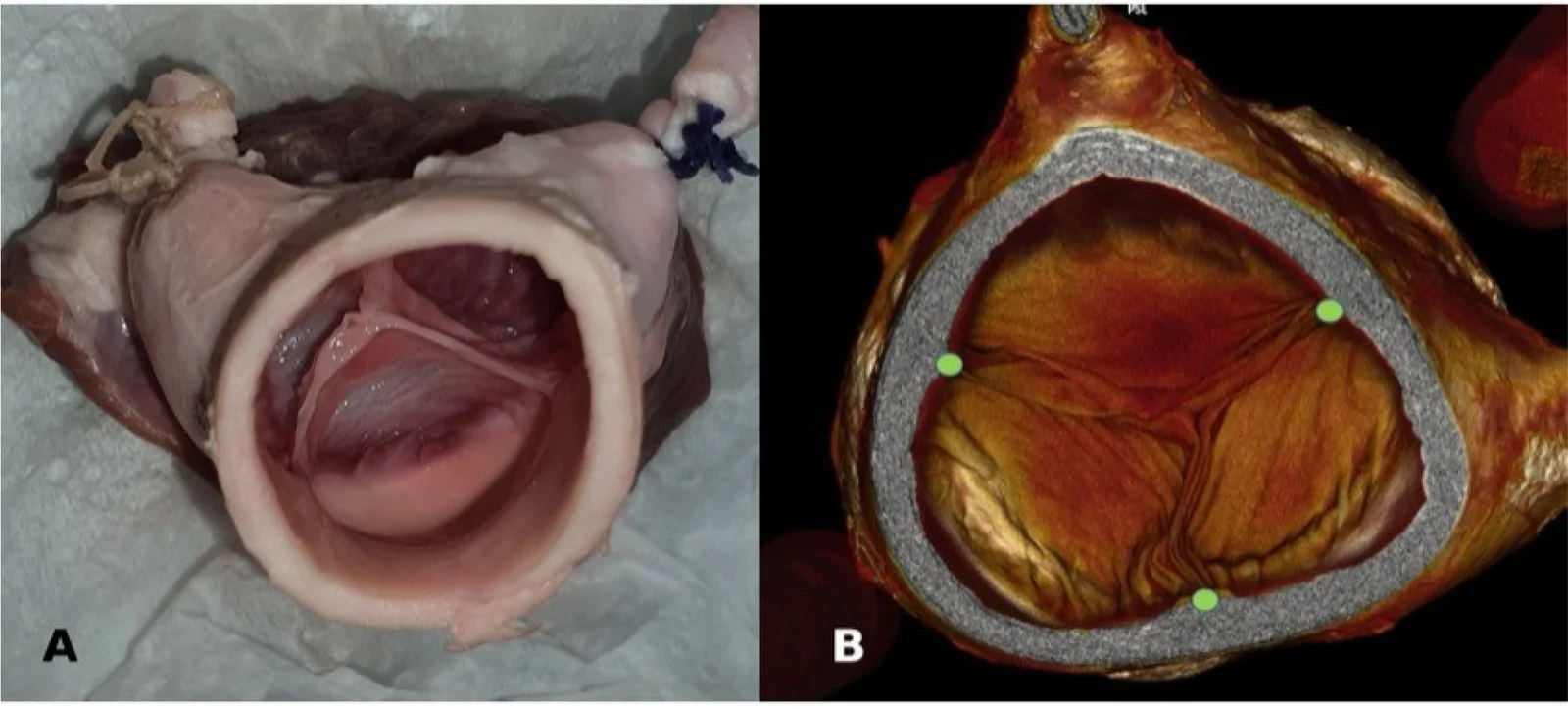
Three geometric landmarks at the upper part of each commissure. (A) Before micro-computed tomography, and (B) after volume rendering images in Horos®. In order to preserve human aortic roots, photos 2A and E2B are taken on pig aortic roots.

**Fig. 3:**
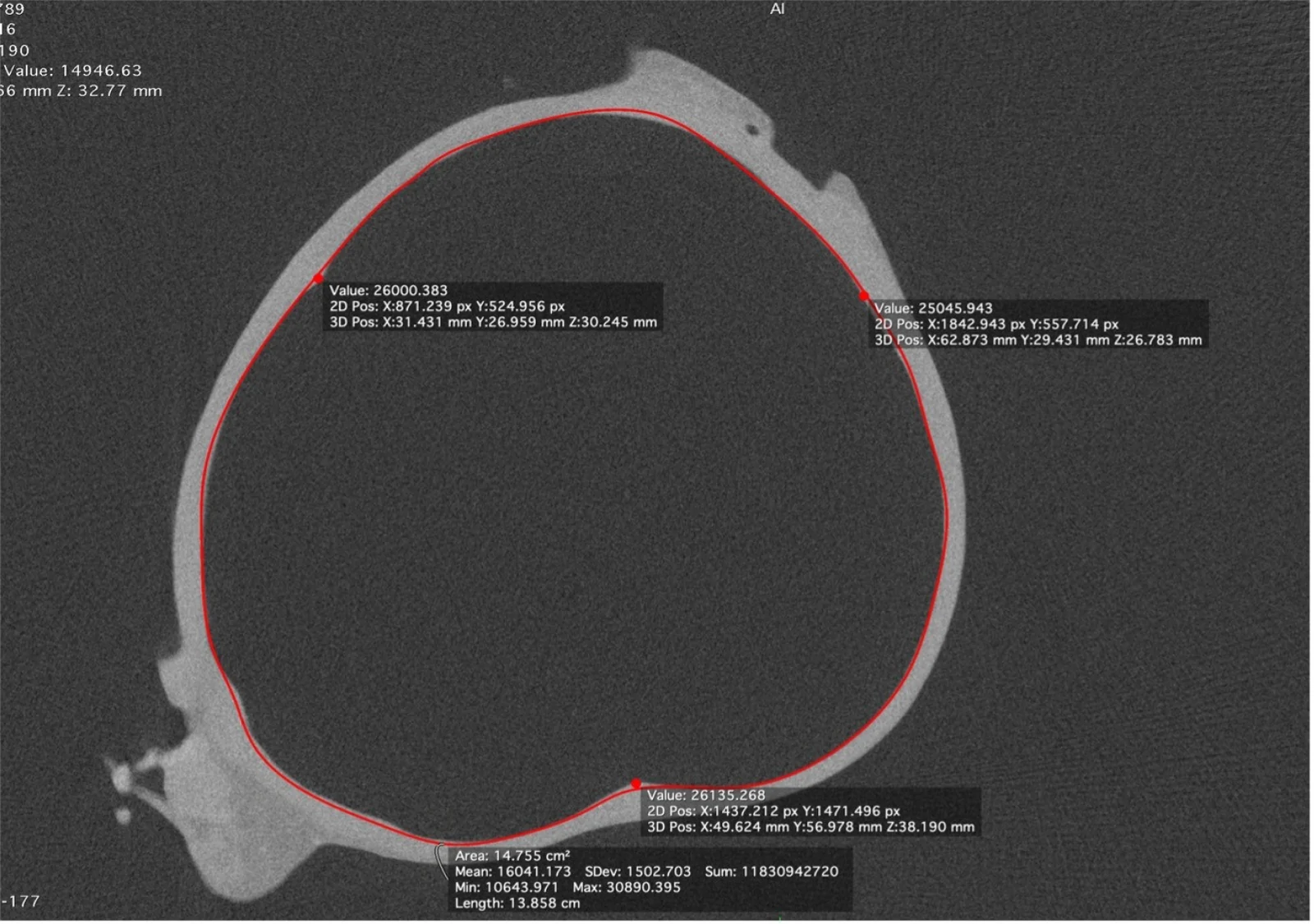
Diameters were obtained from area calculations.

Biomechanical properties, including stress-strain curves and Young’s modulus, were calculated using Laplace’s law. Circumferential stress was determined by considering the internal pressure, radius, and wall thickness, enabling precise quantification of the aortic root’s mechanical response to pressurization.

### Biomechanical Calculations

Circumferential wall stress was calculated using Laplace’s law:

𝞼 = (P x r) / h

where P is internal pressure (Pa), r the internal radius, and h the wall thickness (m). Circumferential strain was defined as the relative change in radius compared with the reference radius at 20 mmHg. Young’s modulus was derived as the slope of the stress-strain curve in its linear region, as also summarized in Table 1.

**Table 1:** Aortic elasticity variable.

Aortic elasticity variable	Formula	Median & IQR Low pressure (20 - 80 mmHg)	Median & IQR High pressure (80 - 120 mmHg)	*P*-value
Compliance (mL/mmHg)	$\frac{D_{Sys}-D_{dia}}{P_{Sys}-P_{dia}}$	0.07 (0.05 - 0.08)	0.01 (0.005 - 0.02)	< 0.001
Distensibility (10^-3^ mmHg^-1^)	$\frac{A_{Sys}-A_{dia}}{A_{dia}\cdot \left(P_{Sys}-P_{dia}\right)}$	5 (3.9 - 5.6)	0.9 (0.43 - 1.19)	< 0.001
Strain (%)	$\frac{A_{Sys}-A_{dia}}{A_{dia}}$	0.29 (0.22 - 0.33)	0.03 (0.02 - 0.05)	< 0.001
Stiffness index	$\frac{Ln\left(P_{Sys}/P_{dia}\right)}{\left(A_{Sys}-A_{dia}\right)/A_{dia}}$	4.7 (4.1 - 6)	12.6 (8.5 - 23.1)	< 0.001
Peterson's elastic modulus (EPeterson) (mmHg)	$\frac{\left(P_{Sys}-P_{dia}\right)}{\left(A_{Sys}-A_{dia}\right)/A_{dia}}$	57 (51 - 74)	333 (226 - 611)	< 0.001
Young's modulus (E) MPa	$\frac{\left(P_{Sys}-P_{dia}\right)\cdot r}{\left(\frac{A_{Sys}-A_{dia}}{A_{dia}}\right)\cdot e}$	0.6 (0.5 - 0.8)	3.2 (2.5 - 5.6)	< 0.001

IQR=interquartile range

Additional morphological measurements, such as wall thickness, were recorded.

### Statistical Analysis

Quantitative data were expressed as medians with interquartile ranges, and categorical variables were reported as frequencies and percentages. Spearman’s rank correlation was employed to explore relationships between biomechanical properties and donor characteristics such as age, weight, and ischemia duration. A *P*-value of < 0.05 was considered statistically significant. Statistical analyses were performed using IBM SPSS Statistics for Windows, version 20 (IBM Corp., Armonk, N.Y., USA).

## RESULTS

### Study Demographics

A total of 10 aortic roots were analyzed. The median donor age was 61.2 years (49.5 - 65.6), with 80% of donors being male. The median time from death to micro-CT imaging was six days (2 - 6.7). Despite this time frame, all samples demonstrated excellent structural preservation, suitable for biomechanical testing and imaging. Donor weight ranged from 45 to 79.7 kg, with a median body mass index (BMI) of 25.4 Kg/m2 (18.7 - 26.9) (Table 2). All donors had died from non-medical causes, specifically either suicide or traffic-related accidents.

**Table 2:** Patient demographics.

Patient demographics	N = 10
Sex	
Male	7 (70%)
Female	3 (30%)
Age, median (IQR) (years)	61.2 (49.5 - 65.6)
Weight, median (IQR) (Kg)	74 (45 - 79.7)
Body mass index, median (IQR) (Kg/m^2^)	25.4 (18.7 - 26.9)
Time between death and micro-CT (days), median (range)	6 (2 - 6.7)

CT=computed tomography; IQR=interquartile range

### Aortic Root Measurements

3D modeling was successfully achieved for all 10 aortic roots, providing high-quality geometrical data. The median diameter of the STJ at 80 mmHg was 32.2 mm (29 - 36). Stress-strain curves obtained from the inflation tests revealed non-linear behavior. At pressures below 80 mmHg, the stress-strain relationship was relatively linear (Figure 4). Beyond 80 mmHg, the curves transitioned into an exponential phase.

**Fig. 4:**
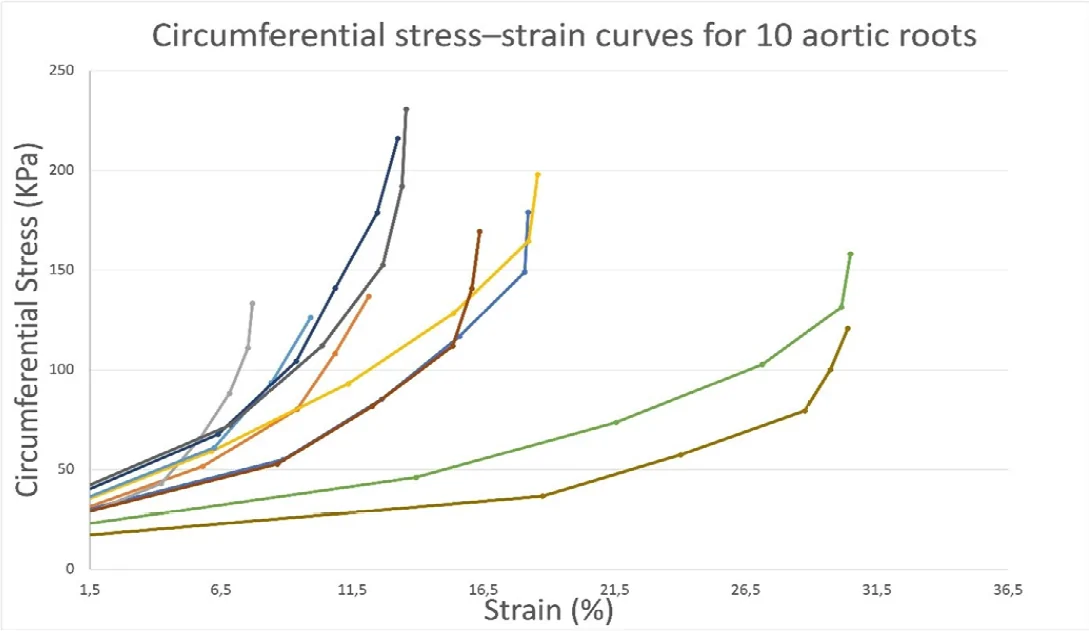
Curve stress/strain for 10 aortic roots.

These parameters exhibited variability, reflecting the inherent anatomical differences among donors. Wall thickness ranged from 1.5 to 2.3 mm.

### Biomechanical Properties

Young’s modulus was significantly lower at low pressures (20 - 80 mmHg) with a median value of 0.6 (0.5 - 0.8) MPa, compared to 3.2 (2.5 - 5.6) MPa at high pressures (80 - 120 mmHg) (*P* = 0.001). Distensibility decreased markedly as pressure increased, with values ranging from 5.6 × 10^-3^ mmHg^-1^ at low pressure to 0.9 × 10^-3^ mmHg^-1^ at high pressure (*P* < 0.001) (Figure 5) (Table 1).

**Fig. 5:**
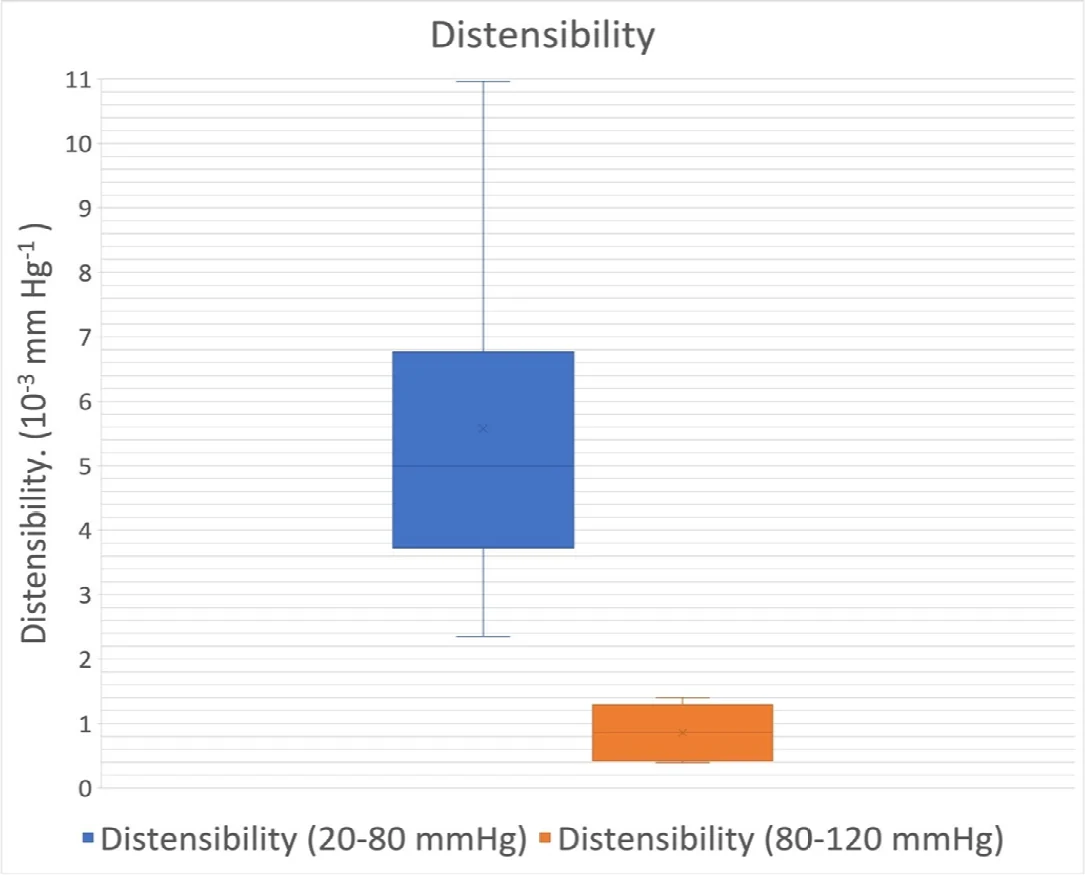
Aortic root distensibility. Between 80 and 120 mmHg (high pressure) and 20 and 80 mmHg (low pressure). Distensibility (10^-3^ mmHg^-1^).

The stiffness index also showed significant variation, increasing from a median of 4.7 at low pressure to 12.6 at high pressure (*P* < 0.001).

### Risk Factors

A significant positive correlation was observed between donor age and Young’s modulus (*Ρ* = 0.76, *P* = 0.011).

In contrast, no significant correlations were found between Young’s modulus and other variables such as BMI (*Ρ* = -0.25, *P* = 0.49), ischemia duration (*Ρ* = 0.25, *P* = 0.49), or STJ diameter at 80 mmHg (*Ρ* = 0.42, *P* = 0.23).

## DISCUSSION

Our findings confirm that the stress-strain relationship of the aortic root exhibits hyperelastic behavior, transitioning from a linear phase at lower pressures (below 80 mmHg) to an exponential phase at higher pressures. This behavior reflects the progressive recruitment of collagen fibers, which contributes to the nonlinear mechanical properties. Specifically, the initial linear phase corresponds to the recruitment of elastic fibers, while the exponential phase is characterized by the straightening of collagen fibers, culminating in increased stiffness. This two-phase behavior aligns with previous studies^[10-16]^ and provides a deeper understanding of aortic biomechanics.

Quantitatively, our results align closely with literature values. For instance, Xuan et al.^[17]^ reported circumferential stress values of 4.2 MPa (2.8-8.8) at high pressures, comparable to our findings. Similarly, Khanafer et al.^[14]^ documented a Young’s modulus of 3.07 MPa, which is consistent with our measurements. The observed decrease in distensibility with age and pressure further supports the well-established principle that the aortic root becomes stiffer over time^[18-20]^. Standard distensibility values, such as 7.4 × 10^-3^mmHg^-1^ for individuals aged 20 - 30 years and 1.8 × 10^-3^mmHg^-1^ for those aged 50 - 60 years, underscore the importance of considering age-related changes in biomechanical analyses^[18,20]^.

In our dataset, age was the only donor-related parameter significantly associated with increased stiffness. A positive correlation was found between donor age and Young’s modulus (*Ρ* = 0.76, *P* = 0.011), indicating that the aortic root becomes progressively less compliant with age, even in the absence of overt pathology. This age-dependent stiffening likely reflects cumulative microstructural remodeling, such as elastin degradation and increased collagen cross-linking. These findings are consistent with established physiological observations^[18,20]^ and reinforce the need to consider donor age when evaluating homografts or modeling patient-specific biomechanical behavior.

Clinically, our findings have significant implications for procedures like the Ross operation, where strict blood pressure control is recommended to prevent neo-aortic root dilation^[21-23]^. By maintaining systolic pressures below 110 mmHg for six - 12 months post-surgery, distensibility can be preserved, potentially enhancing the long-term stability of the autograft. This insight underscores the importance of tailoring postoperative care to optimize biomechanical outcomes.

### Novel Applications of the Method

This innovative technique also holds potential for evaluating new surgical procedures and devices. For example, it could play a critical role in assessing the biomechanical behavior of reconstructed aortic valves, such as those created through the Ozaki procedure. The Ozaki technique, which involves constructing new aortic valve leaflets using autologous pericardium, relies heavily on the biomechanical compatibility of the reconstructed valve with the native aortic root. By providing detailed measurements of stress-strain relationships and geometric changes under varying pressures, this method could offer valuable insights into the durability and mechanical integration of Ozaki valves^[24]^. Furthermore, these data could inform refinements in surgical techniques and material selection, ultimately improving patient outcomes.

Beyond *ex vivo* characterization, the present dataset enables a patient-specific computational workflow to support surgical planning. By coupling blood flow simulation with wall deformation (FSI) and using our pressure-dependent stiffness as material input, preoperative models could predict key hemodynamic outputs after reconstruction (*e.g.*, leaflet kinematics and coaptation, transvalvular gradient, flow symmetry, wall shear stress distribution, and energy loss) together with root deformation under physiological loads. For Ross and Ozaki procedures, such FSI models - augmented with age-dependent stiffness to emulate tissue ageing - may help anticipate long-term behavior (*e.g.*, risk of root dilatation, commissural remodeling, cusp fatigue hotspots), allowing iterative assessment of technical options (leaflet sizing, commissural height, annular stabilization) before surgery. This pathway provides a quantitative framework to optimize surgical design and to estimate durability over time.

### Limitations

Despite its strengths, this study has several limitations. It is an *ex vivo* analysis, which inherently lacks the physiological conditions present *in vivo*. In particular, the use of air as a pressurization medium does not replicate the viscosity or shear forces associated with pulsatile blood flow. While this limitation must be acknowledged, pressure was applied in a static and homogeneous manner, and image acquisition was performed only after full stabilization. Under these conditions, the effect of air compressibility is minimized, and the pressure field can be considered quasi-hydrostatic. This approach was intentionally selected to avoid imaging artifacts related to fluid-based media (*e.g.*, attenuation, bubble formation) and to maintain a dry intraluminal environment that allows high-resolution visualization of wall deformation. We believe this setup provided reproducible and controlled boundary conditions for evaluating radial tissue mechanics.

However, static loading systems have long been used in experimental valve research to isolate and quantify structural responses under controlled conditions^[25]^. Our approach follows this rationale, enabling detailed imaging and mechanical assessment in equilibrium conditions, even if it does not capture flow-related effects.

Additionally, the assumption of a circular cross-sectional geometry at the STJ represents a methodological simplification. Although it does not fully capture the *in vivo* 3D anatomical complexity of the aortic root, this assumption is supported by both anatomical and imaging studies. Cardiovascular magnetic resonance data indicate that the STJ is nearly circular in cross section and relatively uniform in structure^[26]^. Similarly, anatomical reconstructions by Charitos and Sievers^[27]^ describe the STJ as a symmetrical, tubular segment playing a key role in aortic valve competence.

In the present study, the cross-sectional plane was standardized at the level of the three commissural tops, which constitute reliable and unambiguous anatomical landmarks. This standardized approach enabled consistent diameter measurements and a reproducible assessment of wall deformation under static pressurization, while acknowledging the inherent geometric simplification required for inter-specimen comparability.

The one-week postmortem delay constitutes a potential limitation of this study. However, we found no significant correlation between storage duration and the measured Young’s modulus, supporting the reliability of our mechanical assessment.

This is consistent with prior studies. Hemmasizadeh et al.^[28]^ reported a moderate decrease in Young’s modulus (~ 10%) after 48 hours of cold storage at +4°C in porcine aortic tissue, but no further significant degradation up to seven days. These findings suggest that most mechanical changes occur early and tend to stabilize thereafter under refrigerated conditions.

Finally, another limitation of our study is the omission of longitudinal stress analysis. Our focus on radial stress was intentional, as it allowed highly reproducible measurements at a consistent anatomical level - specifically at the height of the commissures, which served as reliable landmarks. In contrast, accurate assessment of longitudinal stress would have required continuous tracking along the vessel’s length, which is more variable due to the lack of stable reference points. While the use of radiopaque or fiducial markers (*e.g.*, beads) could enable such analysis, we opted not to implement them in order to preserve the lumen’s integrity for high-resolution imaging. Finally, our series was limited to ten donor roots, reflecting the scarcity of intact human specimens available for this type of analysis. While this sample size does not allow broad generalization, it supports the feasibility and internal reproducibility of the protocol as a proof-of-concept. Moreover, although the micro-CT protocol requires specialized equipment, it provided consistent and artifact-free reconstructions in all cases. Future studies with larger cohorts and pathological samples will be necessary to confirm the robustness and external validity of these findings.

## CONCLUSION

This study presents a robust *ex vivo* method for simultaneous high-resolution 3D modeling and biomechanical characterization of the aortic root. Our results demonstrated a pressure-dependent increase in stiffness and a nonlinear stress-strain behavior, with Young’s modulus values and stress levels consistent with prior literature. While the method offers excellent spatial resolution and reproducibility, its immediate clinical applicability is limited by its experimental nature. Nevertheless, this protocol provides valuable input data for computational models and offers a reference framework for assessing the mechanical behavior of healthy aortic roots. Future work should explore its application to pathological tissues (*e.g.*, aneurysmal or dissected roots), integrate it into FSI simulations, and correlate biomechanical properties to improve physiological modeling and surgical decision-making.

## Data Availability

The data supporting the findings of this study are available upon request from the authors, subject to applicable ethical, legal, and confidentiality restrictions. To be validated with the responsible parties.

## Artificial Intelligence Usage

The authors declare the use of ChatGPT (GPT-4o, 2025) for English language editing of the manuscript. The content produced by the artificial intelligence tool was reviewed and edited by the authors as necessary, and they take full responsibility for the content to be published.
